# Transient Retention of Photoreceptor Outer Segments in Matrigel-Embedded Retinal Organoids

**DOI:** 10.3390/ijms232314893

**Published:** 2022-11-28

**Authors:** Patricia Berber, Sofiia Bondarenko, Lisa Michaelis, Bernhard Heinrich Friedrich Weber

**Affiliations:** 1 Institute of Human Genetics, University of Regensburg, Franz-Josef-Strauss-Allee 11, 93053 Regensburg, Germany; 2 Institute of Clinical Human Genetics, University Hospital Regensburg, 93053 Regensburg, Germany

**Keywords:** induced pluripotent stem cells, retinal organoids, photoreceptors, outer segments, retinal pigment epithelium, ciliopathy

## Abstract

Retinal organoids (ROs) are three-dimensional retinal tissues, which are differentiated in vitro from induced pluripotent stem cells (iPSC), ultimately forming all main retinal cell types under defined culture conditions. ROs show several highly specialized retinal features, including the outgrowth of photoreceptor outer segments (OSs). In vivo, the photoreceptor OSs are enveloped and maintained by protrusions of retinal pigment epithelium (RPE) cells, the so-called apical microvilli, while ROs fail to recapitulate this critical interaction in culture development. Here, we define specific co-culture conditions aiming to compensate for the missing physical proximity of RPE and OSs in RO development. Accordingly, functional RPE cells and ROs were differentiated simultaneously from the same iPSC clone, the former resulting in byproduct RPE or bRPE cells. While some co-culture approaches indicated a temporary functional interaction between bRPE and RO photoreceptors, they did not improve the photoreceptor histoarchitecture. In contrast, embedding ROs in a basement membrane extract without bRPE cells showed a robust improvement in the rate of photoreceptor OS retention. RO embedding is a quick and easy method that greatly enhances the preservation of photoreceptor OSs, an important structure for modelling retinal diseases with the involvement of photoreceptors.

## 1. Introduction

The study of human retinal development in health and disease is often hindered by the structural and functional complexity of the human retina and a lack of faithful animal or cellular model systems. Although rodent retinae recapitulate many features of their human counterparts, the study of inherited retinal diseases (IRDs) in animal models is particularly challenging due to the genetic and physiological disparity between the species [1]. Cellular model systems can provide a human genetic context, although they often fail to recapitulate the complex multicellular environment in which many diseases arise. For example, retinal ciliopathies are IRDs that directly affect photoreceptors (reviewed in [2,3]), a cell type that in vivo receives intimate support from adjacent retinal pigment epithelium (RPE) cells. The functional relationship between photoreceptors and RPE cells is crucial to retinal health, as the detachment of the RPE from the photoreceptor cell layer in vivo is known to be a serious medical condition, manifesting as retinal detachment or ablatio retinae [4,5].

In recent years, a cellular human model of the retina has been developed, which at least partially recapitulates the multicellular context of this specialized human tissue [6]. Retinal organoids (ROs) are three-dimensional in vitro retinal tissues [7], which are differentiated from stem cells, most commonly from induced pluripotent stem cells (iPSC) [8,9]. To date, a broad variety of differentiation methods have been established, including embryoid body-derived ROs [10], bipotent retinal progenitor cell-derived ROs [11], epithelialized cyst-derived ROs [12], adherent culture-derived ROs [13], and the directed 3D induction of ROs [14].

ROs produce all six main neuroretinal cell types, including the retinal ganglion cells, amacrine cells, bipolar cells, Mueller glial cells, horizontal cells, and photoreceptors [15,16,17,18]. ROs regularly develop ectopic clumps of RPE. ROs generally recapitulate their native retinal spatial organization, and form a ganglion, inner nuclear, and outer nuclear layer [10,19,20,21,22]. Still, there is an important incongruency between ROs and native tissue. The vital interaction between the photoreceptors and RPE is not replicated in ROs (reviewed in [23]). Instead, RO photoreceptors make up the outermost layer of cells and are deprived of the intimate environment created by the RPE cells. Despite the lack of physiological contact with RPE, the RO photoreceptors still develop inner segments (IS), and later even nascent OS [10,24,25].

As retinal ciliopathies directly affect photoreceptor OSs, it is imperative to retain the RO photoreceptor OSs during harvest so that they can be analyzed for phenotypic features of the disease. However, this is challenging, as OSs are fragile and prone to breakage when exposed to shear forces during experimental preparations, e.g., for immunocytochemistry (ICC).

To address this seemingly unavoidable loss, we explored several means of OS stabilization. In vivo, RPE cells envelop and support the photoreceptor OSs, so conceptionally ROs and RPE need to be brought close to each other in co-cultures to attempt to replicate this interaction in vitro. Additionally, ROs were embedded in Matrigel (a soluble basement membrane extract) without the addition of RPE cells to investigate whether this could protect photoreceptor OSs during experimental processing.

## 2. Results

### 2.1. Loss of Photoreceptor OSs during Retinal Organoid Processing

**Figure 1:**
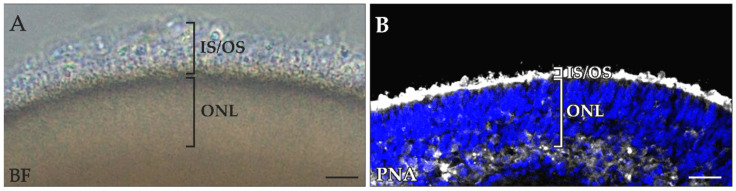
Photoreceptor inner (IS) and outer (OS) segments were lost during retinal organoid (RO) processing. (**A**) Brightfield (BF) image and (**B**) peanut agglutinin (PNA)-positive photoreceptor IS and OS of the same 180-day-old RO after PNA-staining. Prior to processing, ROs revealed IS and OS outgrowth, but upon harvesting and PNA-staining of the RO, the IS/OS layer was considerably reduced, in contrast to the outer nuclear layer (ONL), which remained unaltered in thickness. Counterstaining was performed with 4′,6-Diamidin-2-phenylindol (Dapi) dye. Scale bars: 25 µm.

RO photoreceptor OSs protrude from the RO surface, making them vulnerable to mechanical shearing. Repeatedly, ROs with strong photoreceptor outgrowth lost much of this buildup during harvesting and preparation for ICC experiments (Figure 1A,B). We theorize that the damage was caused by the numerous washing and incubation steps required during sample preparation.

### 2.2. RPE Cells as a Byproduct during RO Differentiation

**Figure 2:**
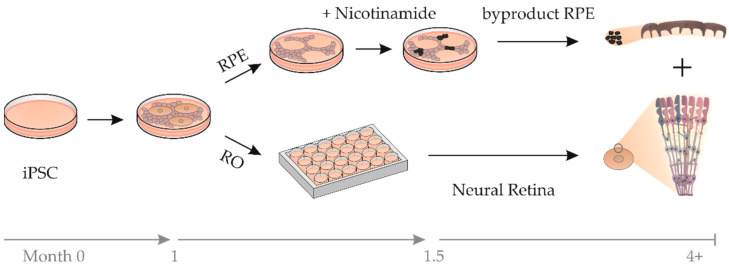
Schematic representation of the simultaneous differentiation of byproduct RPE (bRPE) and retinal organoids (ROs). Induced pluripotent stem cells (iPSCs) were differentiated to ROs and bRPE simultaneously. After 1 month, the ROs were excised and transferred to 24-well plates for further cultivation. The remaining cells were treated with nicotinamide, and after 1.5 months pigmented cell clusters (representing bRPE) were excised and expanded.

To protect and stabilize photoreceptor OSs during RO differentiation, cocultures of ROs with RPE were pursued. We speculated that the likelihood of an efficacious interaction between RO photoreceptors and RPE would increase by utilizing both tissues from a single RO culture with an identical genetic background. A technique was developed to simultaneously differentiate ROs and byproduct RPE (bRPE) (Figure 2). Following existing protocols, ROs are excised from a 2D culture and further cultivated, while the non-RO cells are discarded [10,20]. In our modified protocol, the non-RO cells were treated with nicotinamide for several weeks to reinforce RPE differentiation [26]. Thereafter, the pigmented bRPE clusters were excised and expanded.

**Figure 3:**
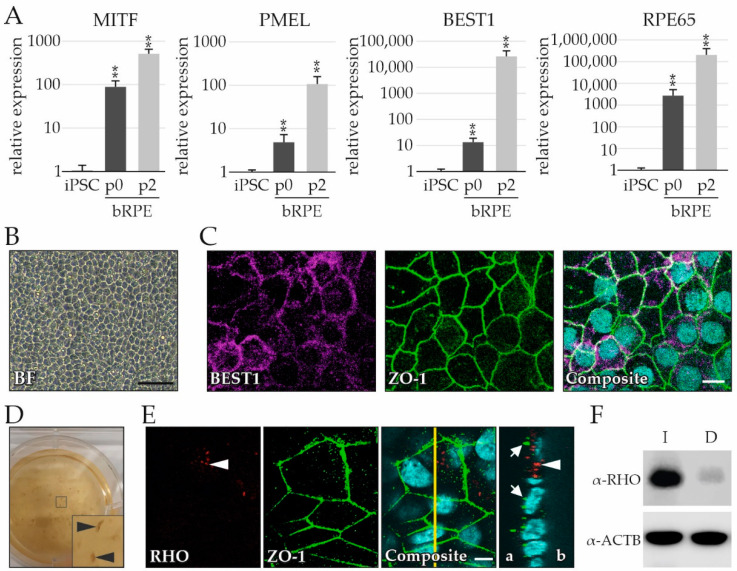
Byproduct RPE (bRPE) cells reveal characteristic retinal pigment epithelium (RPE) expression and typical phagocytotic photoreceptor outer segment (OS) activity. (**A**) Relative expression of RPE markers MITF, PMEL, BEST1, and RPE65 in bRPE cells during differentiation (p0, day 42) and after two passages (p2, day 105), in comparison to undifferentiated induced pluripotent stem cells (iPSCs). (**B**) Brightfield (BF) image of 105-day-old bRPEs exhibiting cobblestone morphology. (**C**) 147-day-old bRPE cells expressing BEST1 (an RPE marker) and ZO-1 (a tight junction marker), forming a honeycomb pattern. (**D**) Pigmented bRPE clusters could be identified during differentiation on day 42. A single well of a 6-well plate is shown. (**E**) After coculture with an RO, the 140-day-old bRPE showed internalization of RHO-positive photoreceptor OS. Z-stacks demonstrate the intracellular localization of RHO-positive OS (the vertical yellow line denotes the localization of the Z-Stack; arrowhead: RHO-positive photoreceptor OS; white arrows: ZO-1-positive tight junctions; a: apical; b: basal). (**F**) Porcine photoreceptor OS internalization by 168-day-old bRPE is indicated by prominent RHO-positive staining (lane labelled I, 37 kDa). Photoreceptor OS degradation by bRPE is indicated by weak RHO-positive staining (lane labelled D). ACTB served as a loading control. Counterstaining was performed with Dapi dye. Scale bars: 10 µm, ** *p* < 0.01 (Bonferroni-corrected).

The bRPE cells were evaluated for expression of RPE markers and recapitulation of characteristics of functional RPE. The mRNA expression of four RPE markers was tested via quantitative real-time reverse transcriptase polymerase chain reaction (qRT-PCR) and included the melanocyte-inducing transcription factor (MITF), the premelanosome protein (PMEL), bestrophin-1 (BEST1), and the retinoid isomerohydrolase RPE65 (RPE65) (Figure 3A). The four markers were highly upregulated during differentiation (p0) and after passaging the bRPE twice (p2) in comparison to undifferentiated iPSCs. The bRPE also exhibited the RPE-typical cobblestone morphology (Figure 3B).

To determine whether bRPE acquired apical–basal polarity, cells were cultured on Transwell filter inserts for 6 weeks and immunostained for BEST1 (localized at the basolateral aspect of the RPE [27,28]) and tight junction protein 1 (ZO-1; localized at the apical aspect of the RPE [29,30]). BEST1 and ZO-1 immunostaining revealed an RPE-typical honeycomb pattern (Figure 3C). bRPE also exhibited strong pigmentation, which was visible even without magnification (Figure 3D).

Finally, the ability of bRPE to phagocytose RO photoreceptor OSs was evaluated. bRPE cells grown on a Transwell filter were cocultured with an RO for two weeks. After the RO spontaneously detached from the bRPE lawn, the bRPE were immunostained with ZO-1 and Rhodopsin (RHO) (Figure 3E). Intracellular RHO-positive photoreceptor OSs were observed (Figure 3E, arrowhead), indicating that the bRPE executed this critical function. To confirm that the bRPE internalize and degrade OSs, bRPE were fed with porcine photoreceptor OSs. The bRPE internalized the OSs, as demonstrated by a prominent RHO signal (Figure 3F, lane labeled I). After four hours of incubation, the bRPE degraded the OSs, as demonstrated by the clear reduction in RHO signal (Figure 3F, lane labeled D).

### 2.3. Testing of Multiple bRPE-RO Coculture Techniques

Several bRPE-RO co-culture techniques were tested for efficiency (Figure S1), with the aim of establishing a robust physical interaction between the bRPE and RO photoreceptors. Each coculture technique was evaluated based on two criteria: (1) the physical proximity of the bRPE to the RO, and (2) indications of a functional interaction between the bRPE and RO photoreceptors.

**Figure 4:**
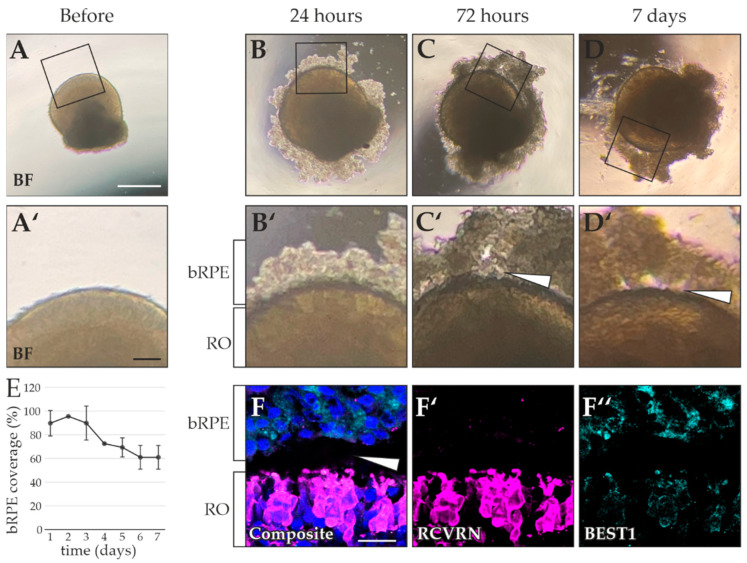
Superficial interaction between byproduct RPE (bRPE) and retinal organoid (RO) photoreceptors. Brightfield (BF) images of the same RO (**A**) before, (**B**) 24 h, (**C**) 72 h, and (**D**) 7 days after coculture with dissociated bRPE. (**A**,**A’**) Prior to coculture induction, no bRPE were located on the RO surface. (**B**) After 24 h, bRPE were observed around the entire RO perimeter. (**B’**) Due to tight adherence of bRPE and RO photoreceptors, close physical proximity was suspected between the bRPE and the RO surface. (**C**) After 72 h, bRPE coverage around the RO had noticeably reduced. (**C’**) Gaps formed between the bRPE and RO surface (arrowhead). (**D**) After 7 days, only minimal bRPE coverage remained. (**D’**) Prominent cavities between bRPE and the RO were observed (arrowhead). (**E**) bRPE coverage of co-cultured ROs decreased over time. (**F,F’,F″**) ICC analysis confirmed the detachment of BEST1-positive bRPE (F″) from the RCVRN-positive photoreceptors (F’) within the RO (F, arrowhead). Counterstaining was performed with Dapi dye. Black squares in A–D indicate the location of the enlarged images shown in A’–D’. RO age: 105–150 days, bRPE age: 105 days. Scale bars: (**A**–**D**) 500 µm, (**A’**–**D’**) 100 µm, (**F**–**F″**) 10 µm.

A first co-culture was induced by adding dissociated bRPE to the RO (RO shown prior to coculture induction in Figure 4A,A’). The coculture was performed with immature ROs that had not yet developed IS and OS outgrowth. The aim was to replicate the in vivo situation, where the neural retina and RPE layer are joined during early development (reviewed in [31]). After 24 h, the bRPE appeared to attach to the RO surface (Figure 4B,B’). After 72 h, some bRPE had detached (Figure C), and a discrete gap was observed between the RO and the remaining bRPE (Figure C’ arrowhead). By day 7, most of the bRPE had detached from the RO surface (Figure 4D), with prominent parting of the bRPE cells from the RO (Figure 4D’ arrowhead). Overall, bRPE coverage decreased steadily over time (Figure 4E). Of note, the bRPE coverage that remained after 7 days (61 ± 0.1%) did not appear to adhere to the RO and had formed noticeable gaps. These gaps were confirmed by immunostaining, where recoverin (RCVRN)-positive photoreceptors and BEST1-positive bRPE cells showed a clear separation between the two tissues (Figure 4F,F’,F’’; arrowhead). Furthermore, there were no substantial indications of a functional interaction between the bRPE and RO, as no robust RCVRN internalization by the bRPE was observed (Figure 4F,F’,F″ show a lack of RCVRN signal within the bRPE).

Next, cocultures were treated with additives to improve bRPE attachment, and harvested after 48 h, as previous experiments suggest that longer coculture duration leads to bRPE detachment. Neither the addition of an apoptosis inhibitor (ApInh) nor the addition of soluble components of the photoreceptor extracellular matrix (PrECM) improved bRPE attachment (Figure S2A–F).

**Figure 5:**
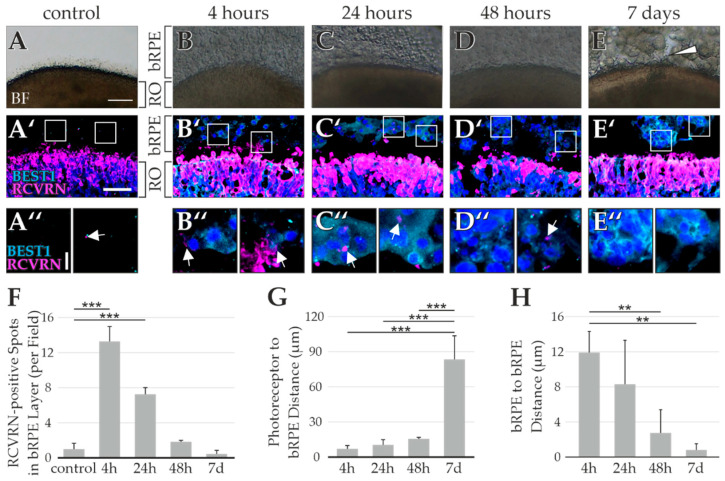
Cocultures of retinal organoids (RO) with byproduct RPE (bRPE) in Matrigel suspension indicate a transient functional interaction. Brightfield (BF) images are shown of (**A**) a control RO that was not suspended in the bRPE–Matrigel solution, and of cocultures after (**B**) 4 h, (**C**) 24 h, (**D**) 48 h, and (**E**) 7 days. bRPE cells were initially close to the RO, but after 1 week cavities were observed between the RO and bRPE (**E**, arrowhead). (**A’**) In the control ROs, no BEST1-positive bRPE were seen outside the RCVRN-positive RO outer nuclear layer. After co-culturing for (**B’**) 4 h, (**C’**) 24 h, and (**D’**) 48 h, BEST1-positive bRPE were located near RCVRN-positive photoreceptors. (**E’**) After 7 days there were only few bRPE aggregates close to the RO photoreceptors. (**A″**) In controls, prominent RCVRN staining was not observed outside the RO. Rarely, some discrete RCVRN-positive signals were seen (white arrow), which were judged likely unspecific. Co-cultures of (**B″**) 4 h and (**C″**) 24 h showed RCVRN uptake by the bRPE, indicting a functional interaction (white arrows). (**D″**) After 48 h, the RCVRN uptake had noticeably decreased. (**E″**) At day 7, no RCVRN uptake by the bRPE was noticed. (**F**) The co-cultures analyzed after 4 or 24 h had significantly more RCVRN-positive staining outside the RO than the control ROs, indicating that the bRPE had taken up RCVRN signals from the RO. (**G**) The bRPE were initially close to the RO photoreceptors, but migrated away from the RO after 7 days. (**H**) In contrast, the distance between individual bRPE cells steadily decreased over time, as the bRPE formed aggregates within the Matrigel. RO age: > 180 days, bRPE age: 119 days. Scale bar (**A**–**E**) 100 µm, (**A’**–**E’**) 50 µm, (**A″**–**E″**) 10 µm. White squares in E’–D’ indicate the location of the enlarged images shown in (**A″**–**E″**). ** *p* < 0.01, *** *p* < 0.001 (Bonferroni-corrected).

The co-culture was further modified with the aim of stabilizing bRPE-RO interaction. Accordingly, the dissociated bRPE were suspended in Matrigel, and the ROs were coated in the bRPE–Matrigel solution. Older ROs (time in culture at least 180 days) were used, which had already developed IS and OS outgrowth (as seen in the control RO, which was not embedded in bRPE–Matrigel, Figure 5A), since we theorized that this could improve bRPE interaction. Brightfield microscopy of co-cultures after 4 h, 24 h, and 48 h (Figure 5B–D, respectively), showed that bRPE cells were in close physical contact to the ROs at each timepoint. Co-culturing for 7 days, however, revealed discrete cavities between the bRPE and the RO perimeter (Figure 5E arrowhead). ICC stained RCVRN-positive photoreceptors in all ROs (Figure 5A’–E’), while cocultured ROs revealed BEST1-positive bRPE cells in their immediate neighborhood (Figure B’–E’). While the bRPE were initially evenly distributed within the Matrigel (Figure B″), they began forming aggregates over time (Figure C″,D″). After 7 days the bRPE had formed compact multicellular clusters (Figure 5E″).

RCVRN uptake by bRPE was observed after 4 and 24 h (Figure 5B″,C″, respectively, white arrows), likely indicating a functional interaction between the photoreceptors and bRPE cells. After 48 h the RCVRN uptake by bRPE had markedly decreased (Figure 5D″) and was virtually non-existent after 7 days (Figure 5E″). This effect was quantified by counting the RCVRN-positive spots located in the bRPE layer, which showed a significant increase after 24 and 48 h in comparison to the control ROs, which were not embedded in the bRPE–Matrigel mixture (Figure 5F). Of note, the amount of RCVRN-positive staining in ROs embedded in Matrigel (without bRPE) were also counted and were comparable to the control ROs (1 ± 0.7 spots per field in the control ROs vs. 1.4 ± 0.7 in the ROs embedded exclusively in Matrigel).

The distance between the RO photoreceptors and bRPE cells was also quantified, demonstrating that the bRPE cells were close to the ROs for the first 48 h, but migrated away between the 2nd and 7th day in culture (Figure 5G). Furthermore, the distance between individual bRPE cells decreased over time (Figure 5H), indicating that the bRPE gradually began to aggregate within the Matrigel, and eventually formed tight multicellular clusters. Taken together, it appears that the bRPE internalized RCVRN signals while they were close to the RO photoreceptors, but over time they detached from the RO, thoroughly digesting the initially internalized RCVRN, but failing to internalize new RCVRN. These findings indicate a transient, functional relationship for this co-culture condition.

Nevertheless, we failed to obtain evidence that the co-culture improved outer segment retention in the ROs. The grainy texture of the bRPE–Matrigel suspension impeded pristine cryosectioning, which was confirmed by the slightly disrupted photoreceptor histoarchitecture seen in the RCVRN immunostainings (Figure 5B’). We therefore decided against further in-depth analyses of photoreceptor outer segments in co-cultured ROs.

Additional co-culture techniques were performed with the aim of producing a more robust and sustained functional interaction. For example, ROs were co-cultured with dissociated bRPE for 48 h and then embedded in Matrigel. Although the bRPE were observed around the RO perimeter prior to embedding (Figure S1G), the bRPE detached during this phase (Figure S1H). To determine whether polarized bRPE could improve interaction, ROs were added to bRPE grown on Transwell filters. Despite most careful handling, the ROs invariably detached from the bRPE lawn (Figure S1I,J). Finally, Matrigel or Hydrogel without bRPE cells was added to the co-cultures on Transwell filters [32]. Still, the addition of the gels caused the RO to separate from the bRPE.

### 2.4. RO Embedding Improves OS Retention

**Figure 6:**
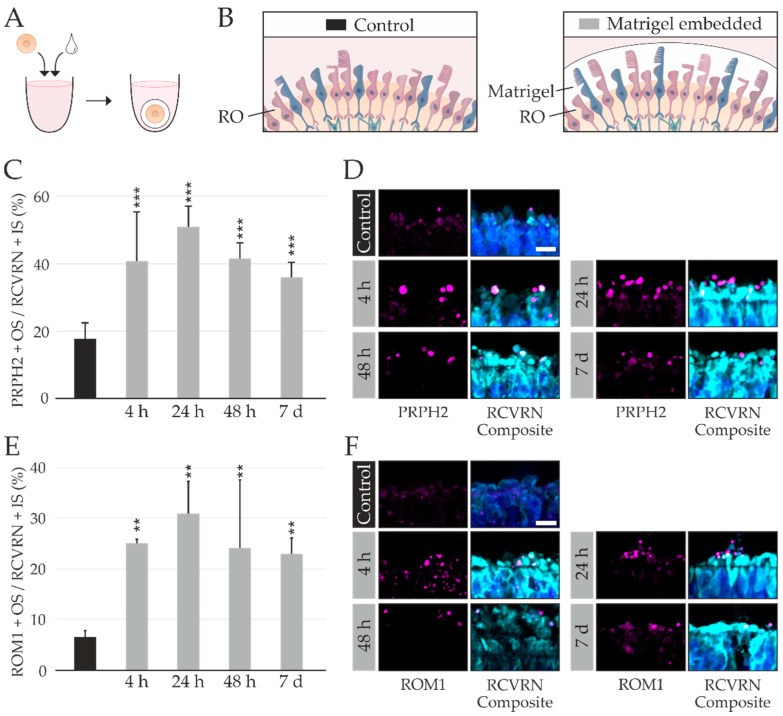
Matrigel embedding protected photoreceptor outer segment (OS) histoarchitecture. (**A**) Diagram of the Matrigel (represented by a white droplet) embedding procedure. (**B**) Schematic representation of photoreceptor OS loss during processing without Matrigel embedding (left image), and improved photoreceptor OS retention in Matrigel-embedded retinal organoids (ROs) (right image). (**C**) The percentage of PRPH2-positive photoreceptor OS relative to the number of RCVRN-positive photoreceptor inner segments (IS) is shown. ROs embedded in Matrigel for 24 h showed the highest proportion of photoreceptors with preserved OS (51 ± 6.1% vs. 17.8 ± 4.6% in control ROs, *p* = 0.0002). (**D**) Exemplary PRPH2- and RCVRN-immunostained images quantified in (**C**). (**E**) The percentage of ROM1-positive photoreceptor OS relative to the number of RCVRN-positive photoreceptors IS is shown. ROs embedded in Matrigel for 24 h showed the highest proportion of photoreceptors with preserved OS (30.9 ± 6.4% vs. 6.6 ± 1.2% in control ROs, *p* = 0.003). (**F**) Exemplary ROM1- and RCVRN-immunostained images quantified in (**E**) are shown. Counterstaining was performed with Dapi dye. Scale bars: 10 µm, ** *p* < 0.01, *** *p* < 0.001 (Bonferroni-corrected).

Although some indication of a functional interaction had been observed in the bRPE-RO co-culture, the interaction did not benefit the histoarchitecture of the RO photoreceptors (especially the OSs). Therefore, a further approach was tested, specifically meant to stabilize the RO photoreceptor OSs. This approach did not require a co-culture with bRPE. Here, the ROs were embedded directly into Matrigel to protect the OSs from shear forces during harvest (Figure 6A,B). The embedded ROs were harvested after 4 h, 24 h, 48 h, or 7 days. Control ROs were not embedded in Matrigel.

To ascertain whether Matrigel embedding improved OS retention, the number of peripherin 2 (PRPH2)-positive OSs relative to the number of RCVRN-positive ISs was determined. Embedded ROs retained more of their photoreceptor OSs than the control ROs, indicating a robust improvement in stabilizing OSs during processing (Figure 6C,D). The highest rate of PRPH2-positive OS retention was observed in the ROs embedded for 24 h (51.0 ± 6.1% vs. 17.8 ± 4.6% in the control ROs, *p* = 0.0002), while OS retention decreased over time in ROs embedded for 7 days (7 days vs. 24 h: 36.1 ± 4.4% vs. 51.0 ± 6.1%). This suggests that Matrigel embedding may not be suitable for a long-term culture. Of note, the histoarchitecture of the ROs embedded in pure Matrigel (Figure 6D,F) was preserved better than that of the ROs embedded in the bRPE–Matrigel mixture (Figure 5A’,B’). This may be due to suboptimal cryosectioning conditions, as the grainy texture of the bRPE–Matrigel mixture prevented pristine sectioning.

To confirm improved OS retention by Matrigel embedding, the number of retinal outer segment membrane protein 1 (ROM1)-positive OSs relative to the number of RCVRN-positive ISs was determined (Figure 6E,F). Again, the highest rate of retention was observed in the ROs embedded for 24 h (30.9 ± 6.4% vs. 6.6 ± 1.2%, *p* = 0.003), and the ROs embedded for 7 days had a lower rate of retention (23.0 ± 3.1%). Overall, embedding ROs in Matrigel for 24 h is a fast and effective method to protect OSs during harvest.

## 3. Discussion

Establishing ROs as retinal disease models with broad applications in basic research and therapeutic use greatly depends on an improved modulation of the in vitro situation to closely resemble in vivo conditions. In this study, we aimed to improve the architectural preservation of RO photoreceptor OSs. Our results suggest that bRPE–RO co-cultures exhibited a functional (albeit temporary) interaction between the bRPE and RO photoreceptors, as would be a prerequisite for metabolic homeostasis at the regular RPE-neural retina interphase. Overall, we were successful in shielding the OSs during processing, as Matrigel embedding of ROs for 24 h doubled the amount of photoreceptor OSs retained during harvesting without requiring a co-culture technique with bRPE. Research into retinal ciliopathies in an RO model system could greatly benefit from the embedding technique shown here, as it is straightforward, requires minimal hands-on time, and shows a better preservation of RO histoarchitecture than known co-cultures with bRPE.

RO photoreceptor OSs are of wide interest within the RO research community. West et al. recently published a modified differentiation protocol, which improved the development of RO photoreceptor OSs [33]. This protocol resulted in impressive OS outgrowth. Still the OSs were exposed to shear forces during culture and harvest. In fact, all current RO differentiation protocols produce ROs in which the OSs protrude from the RO surface (reviewed in [23]), eventually exposing them to shear forces. A combination of the West et al. differentiation protocol and our embedding technique may further improve OS development and retention, thereby integrating both accomplishments in order to achieve the overarching goal of building a retinal model system even closer to the in vivo situation.

In theory, the embedding technique developed in this study could be modified to further stabilize OSs during development, not just during harvest. In this study, OS retention decreased over time between time points 24 h and 7 days, indicating that the Matrigel coating may have impeded nutrient accessibility to the RO. Embedding the ROs in a thinner layer of Matrigel, however, had a greater likelihood that the bRPE cells escaped the Matrigel shell. Future studies need to test whether ROs could be embedded in a different scaffolding substance than Matrigel for a far longer time period. We used Matrigel as it provides an ideal transition from initially liquid to a solidifying state to a gel consistency, while still being conducive to cryosectioning. Other substances were considered at the outset, but they either solidified too quickly (Geltrex™, Life Technologies, Carlsbad, CA, USA), or failed to provide the appropriate consistency for cryosectioning (HyStem^®^-C, Sigma-Aldrich, St. Louis, MO, USA). Overall, Matrigel embedding is an effective and fast way to improve OS retention during harvest, which we anticipate will be highly interesting for other research groups focused on RO biology. Still, future studies may modify our method to shield the OSs long-term, e.g., during developmental phases of RO culturing.

Despite numerous approaches, co-culturing ROs and bRPE showed at best a transient interaction between the bRPE and RO photoreceptors. Based on prior criteria, we endeavored to investigate success of the co-culture (physical proximity and functional interaction); the coculture with bRPE suspended in Matrigel was the most promising, as the bRPE were initially close to the photoreceptors, and appeared to take up RCVRN signals. Nevertheless, the bRPE detached or migrated away from the RO and stopped internalizing RCVRN after only a few days. In the co-culture without Matrigel, the dissociated bRPE initially appeared close to the RO, but detached over time and did not show signs of RCVRN uptake. Numerous modifications to the co-culture techniques failed to notably improve the co-culture success, which caused us to reflect on and compare the co-cultures to the native situation.

In vivo, RPE cells envelop photoreceptor OSs with multiple protrusions known as microvilli [34]. As iPSC-derived RPE were shown to greatly mimic cellular features of in vivo RPE in terms of morphology, pigmentation, mRNA expression, and function [27,35], it is reasonable to assume that the proper interaction between these two differentiated cell types should also produce a stable interface in vitro. It is unlikely that insufficient bRPE quality could have caused the cells to detach from the RO surface, as the bRPE expressed known RPE markers and showed the uptake and degradation of OS. The maturation stage of the ROs and bRPE is also an unsatisfactory explanation, as a wide time window of maturation stages was examined in the present study. Instead, it may be crucial to consider the time period where the bRPE and OSs were close to each other in vitro. Possibly, the developmental window or the duration of interaction was too short to allow the bRPE to develop functionally relevant microvilli.

It is curious that co-culture with bRPE suspended in Matrigel showed within only a few days that the bRPE moved away from the RO. It is possible that the phagocytosis of photoreceptor OSs (although a functional hallmark of RPE in vivo) is a challenge for the bRPE in vitro to make them migrate away from their stressor. Although previous studies have examined co-cultured RPE and ROs, they did not focus on the physical interaction between the two cell types [32,36], while the data we present here indicate that initiating a robust interaction in vitro is far from the in situ situation. Nevertheless, although only for a transient time, we observed what appeared to be a functional interaction between the bRPE and RO photoreceptors, although definite proof of an intimate interaction remains to be shown. Future studies need to focus on the establishment of a rather long-term interaction between the bRPE and RO, therefore increasing the likelihood of a durable functional relationship. Overall, the replication of RPE and photoreceptor interaction in vitro is proving demanding, even though this is a critical intercellular relationship within healthy retina. An in vitro replication of this crucial interaction would likely alleviate the use of ROs for research and spearhead new avenues of investigation.

## 4. Materials and Methods

### 4.1. Sampling of Biomaterials, iPSC Reprogramming and Maintenance

Three iPSC lines, reprogrammed from peripheral blood mononuclear cells (PBMCs) or dermal fibroblasts, were obtained from adults with no history of retinal disease after informed consent and approval of the study by the local ethics committee (Reference No. 11-101-0228). Two clones per iPSC line were used. Fibroblasts and PBMCs were reprogrammed according to Ref. [27] and Ref. [37].

iPSCs were maintained in mTeSR^TM^ Plus medium (Stemcell Technologies, Cologne, Germany) with 25 µg/mL Gentamycin (Merck KGaA, Darmstadt, Germany) on plates coated with hESC-qualified Matrigel^®^ (Corning, New York, NY, USA), and cryopreserved in CryoStor^®^ CS10 medium (Stemcell Technologies).

### 4.2. Differentiation and Characterization of RO and bRPE

ROs were differentiated according to Method 3 (M3) in Ref. [20], modified after [10,25]. Upon excision of the retinal domains on day 21–25, the remaining cells were used for bRPE differentiation. Maintenance of bRPE cells was in the same medium used for stage-specific RO culturing: retinal differentiation medium (RDM) until day 42, retinal cup medium 2 (RC2) until day 90, and retinal cup medium 1 (RC1) thereafter, medium composition provided in Ref. [20], with the addition of 1.2 mg/mL nicotinamide (Merck KGaA). On day 42, strongly pigmented cells were identified and mechanically isolated using cannulas (Becton Dickinson, Franklin Lakes, USA) under an inverted brightfield microscope (Leica S6 D, Leica Microsystems GmbH, Wetzlar, Germany), followed by incubation in TrypLE^TM^ Select (Life Technologies, Carlsbad, CA, USA) at 37 °C for 15 min in a water bath. The bRPE were centrifuged at 300 relative centrifugal force (rcf) for 5 min and the supernatant was removed. The bRPE were resuspended in maintenance medium supplemented with 1.2 mg/mL nicotinamide and cultured on plates coated with Matrigel^®^ Growth Factor Reduced Basement Membrane Matrix (Matrigel GFR, Corning) until day 75 (±5 days). The cells were passaged 1:3 or 1:6 onto Matrigel GFR coated plates using TrypLE^TM^ Select. On day 105 the bRPE were cryopreserved in CryoStor^®^ CS10 medium.

Prior to ICC the bRPE were thawed, seeded on 6-well Matrigel GFR coated plates, and cultured for two weeks. For ICC, the bRPE were then passaged onto Transwell filters (ThinCert^®^ Cell Culture Inserts, Greiner Bio-One, Kremsmünster, Austria) and cultured for 6 weeks. Brightfield images were taken with a Nikon Eclipse TE2 microscope (Nikon Instruments Europe BV, Amstelveen, the Netherlands).

### 4.3. RNA Isolation and Reverse Transcription

RNA was isolated from undifferentiated iPSC, bRPE samples on day 42 (p0), and on day 105 (p2). Samples were lysed with QIAshredder^®^ columns (Qiagen GmbH, Hilden, Germany) or with a Tissue Lyser II (Qiagen) and a clean metal bead. RNA was isolated using the PureLink™ RNA Mini Kit (Invitrogen, Carlsbad, CA, USA), and DNase digestion was conducted with RNAse-Free DNAse (Qiagen) diluted 1:8 in Buffer RDD (Qiagen). RNA concentrations were measured on a NanoDrop^®^ ND1000 Spectrophotometer (NanoDrop, Wilmington, Germany, USA). Reverse transcription was performed with the RevertAid Reverse Transkriptase (Thermo Fisher Scientific, Waltham, USA) and Random Hexamer Primers (Thermo Fisher Scientific) using 1mM dNTPs (Genaxxon, Ulm, Germany) according to the manufacturer’s instructions. cDNA samples were diluted to 20 ng/µL in RNAse-free H_2_O and stored at −20 °C.

### 4.4. qRT-PCR

The reaction mixtures for qRT-PCR contained Takyon™ ROX Probe 2X MasterMix dTTP (Eurogentec, Seraing, Belgium), 1 µM KiCqStart™ primers (Merck KGaA), 312.5 nM KiCqStart™ probes (Merck KGaA), and 50 ng of cDNA. Samples were run in technical duplicates or triplicates in MicroAmp™ Optical 384-Well Reaction Plates (Life Technologies) on a QuantStudio^®^ 5 Real-Time PCR Systems (Life Technologies).

Data were analyzed according to the ΔΔCt method [38]. Measurements with a SD greater than 0.4 Ct values were excluded. Expression was first normalized to the expression of housekeeper gene hypoxanthine phosphoribosyltransferase-1 (HPRT1), followed by normalization to the mean Ct value in iPSC samples. To test for significance, a one-way ANOVA test, post hoc Tukey test, and Bonferroni correction for multiple comparisons were performed [39,40].

### 4.5. Immunocytochemistry

For ICC, the ROs were rinsed in Dulbecco‘s phosphate-buffered saline (DPBS, Life Technologies), and fixed in 4% paraformaldehyde (PFA; Thermo Fisher Scientific) for 30 min. The fixed ROs were washed three times in DPBS for 5 min and incubated in 6.75% sucrose (Merck KGaA) in DPBS (*w*/*v*) for 1 h at RT, followed by another hour at RT in 12.5% sucrose, which was then replaced by 25% sucrose. The samples were incubated overnight at 4 °C. The next day, the 25% sucrose was replaced by 1:1 Epredia™ Neg-50™ Frozen Section Medium (Fisher Scientific GmbH, Schwerte, Germany) and 25% sucrose and incubated for 1 h at RT. Samples were rinsed briefly in Neg-50^TM^ and transferred to single use embedding molds in fresh Neg-50^TM^. The embedded ROs were frozen at −20 °C and transferred to −80 °C for long term storage. The entire RO was cryosectioned into 10 µm sections and the sections were distributed on 8 SuperFrost Plus Adhesion Slides (VWR International BVBA, Leuven, Belgium).

bRPE on Transwell filters and RO (with or without bRPE) cryosections (10 µm) were fixed and immunostained according to Ref. [20]. The following primary antibodies and dilutions were used: BEST1 1:500 (ab2182, Abcam, Cambridge, UK), PRPH2 1:2 (gift from Robert S. Molday, University of British Columbia, Vancouver, BC, Canada), RCVRN 1:2000 (AB5585, Merck KGaA), RHO1D4 1:1000 (gift from Robert S. Molday, University of British Columbia, Vancouver, BC, Canada), ROM1 1:2 (gift from Robert S. Molday, University of British Columbia, Vancouver, BC, Canada), ZO-1 1:500 (61-7300, Life Technologies). For samples stained with PNA, Alexa Fluor^®^ 594 goat anti-mouse IgG was replaced with 4% Alexa 594-conjugated peanut agglutinin (Invitrogen).

### 4.6. Photoreceptor OS Phagocytosis Assay

Photoreceptor OSs were isolated from porcine retinae according to Ref. [27]. Their concentration was determined by sucrose density gradient centrifugation according to Refs. [27,41], and the samples were diluted according to Ref. [41]. The assay was conducted with bRPE grown on 6-well Transwell filter inserts for 6 weeks. The porcine OSs were passed through a 40 µm filter and suspended in OptiMEM™ (Thermo Fisher Scientific). bRPE were incubated in the OS solution for 2 h. After removal of the solution, the bRPE were washed three times with DPBS and incubated in maintenance medium for 2 h. Samples for Western blot analysis were taken to ascertain the internalization of OSs by the bRPE. After 4 h, additional samples were taken to investigate the degradation of OSs by the bRPE. Samples were harvested according to ref. [41] with one modification: samples were heated to 95 °C for 10 min instead of 5 min. Western blotting and analysis were performed according to Ref. [41].

### 4.7. bRPE and RO Coculture Techniques

Multiple bRPE–RO co-culture techniques were tested including (1) co-cultures with dissociated bRPE; (2) co-culture with dissociated bRPE, treated with ApInh or PrECM; (3) co-culture with bRPE suspended in Matrigel GFR and RO; (4) co-culture with dissociated bRPE and RO, suspended in Matrigel GFR; (5) co-culture with bRPE grown on Transwell filters; and (6) co-culture with bRPE grown on Transwell filters stabilized with ECM-like compounds. bRPE and ROs were derived from an identical iPSC line, respectively.

Co-cultures were induced with juvenile and mature ROs and bRPE, in an attempt to induce a functional relationship between the two tissues. Co-cultures (1) and (2) were induced with ROs that had not yet developed ISs and OSs (at least day 105, but no older than day 150) and 105-day-old bRPE. The ROs used in co-cultures (3)–(6) were at least 180 days old. The bRPE used for co-cultures (3) and (4) were cryopreserved on day 105, thawed and cultured on 6-well Matrigel–GFR-coated plates for two weeks prior to coculture induction. The bRPE used for co-cultures (5) and (6) were cryopreserved on day 105, thawed, and cultured on Matrigel–GFR-coated Transwell filter inserts for 6 weeks prior to co-culture induction. Before utilizing bRPE cells in the co-culture experiments, they were weaned off nicotinamide over three medium exchanges.

Specifics to the various coculture techniques tested:

(1) bRPE cells were dissociated with TrypLE^TM^ Select and suspended in maintenance medium without nicotinamide [20]. The bRPE were counted using a CASY^®^ Cell Counter and Analyzer (Innovatis Roche AG, Bielefeld, Germany). One RO and 100,000 bRPE cells were added to one well of a Costar^®^ Ultra-Low Attachment 96-Well Plate with round bottoms (Corning). Co-cultures were imaged after 1 day, 3 days, and 7 days using a Nikon Eclipse TE2 microscope. After 14 days, the co-cultures were cryosectioned and immunostained as described previously.
(2) Co-culture was performed as described in (1) and treated with the following additives, which were applied when the bRPE were introduced to the RO. Co-cultures were treated with ApInh: 5 µM Blebbistatin (Cayman Chemical, Michigan, USA) or with PrECM: 5 µL/mL Fibronectin (Life Technologies), 47.5 µg/mL Laminin (Cayman Chemical), and 16.6 µL/mL vitronectin (kindly provided by Fabiola Biasella University of Regensburg, Regensburg, Germany and purified according to [42]). The PrECM components were chosen based on Ref. [43]. The co-cultures were harvested after 48 h, cryosectioned and immunostained as described previously.
(3) For the co-culture with bRPE suspended in pure Matrigel GFR, 500,000 bRPE were dissociated using TrypLE^TM^ Select and suspended in 10 µL cold Matrigel GFR (based on ref [44]). The RO was transferred to sterile Bemis™ Parafilm™ M Laboratory Wrapping Film molds (Fisher Scientific GmbH), embedded in the bRPE–Matrigel GFR mixture and incubated for 20 min at 37 °C. After 20 min 10 µL cold Matrigel GFR was added, and the cultures were incubated at 37 °C for 10 min. ROs were harvested after 4 h, 24 h, 48 h, or 1 week. Control ROs were not embedded in the bRPE–Matrigel mixture.
(4) Co-culturing was conducted as described in (1) and incubated for 48 h. Brightfield images were taken using a Nikon Eclipse TE2 microscope. The co-culture was transferred to sterile Bemis™ Parafilm™ M Laboratory Wrapping Film molds, embedded in the Matrigel GFR mixture and incubated for 30 min at 37 °C. After embedding, brightfield images were taken using a Nikon Eclipse TE2 microscope.
(5) For the co-cultures on Transwell filter inserts, bRPE were cultured as described previously. The ROs were gently added to the Transwell filter inserts and incubated for at least 2 days. The ROs spontaneously detached from the inserts while handling, during medium changes, and/or during fixation in 4% PFA. After detachment, the Transwell filter inserts were immunostained to analyze OS uptake by the bRPE.
(6) Optionally, 200 µL Matrigel GFR (Corning, New York, USA) or 200 µL hyaluronic acid-based hydrogel HyStem^®^-C (Sigma-Aldrich, St. Louis, USA) was added after plating the ROs on the bRPE lawn (based on ref. [32]).

### 4.8. Quantification of bRPE Coverage

To quantify bRPE coverage around ROs, brightfield images of co-cultures were imported into CorelDraw (Corel Corporation, Ottawa, Canada). Coverage was analyzed using the Ellipse tool, in the circle setting. The start and final angles were used to calculate the percentage of the RO, which was covered by bRPE. Areas were only considered to be bare if there were no bRPE present. Areas with gaps were included in the coverage measurement.

### 4.9. Interaction Analysis of bRPE-RO Cocultures

Immunocytochemistry, microscopy, and analysis was performed with ROs embedded in bRPE–Matrigel and control ROs in parallel. Two to five ROs were investigated per condition (overall average 3.2 ± 1.3). Three images were taken per RO at 10× magnification with an Olympus Fluoview FV3000 confocal microscope (Olympus Life Sciences, Hamburg, Germany). RCVRN-positive spots in the bRPE layer were manually counted using the cell counter plugin in Fiji [45]. For the control ROs that did not contain a bRPE layer, the RCVRN-positive spots outside the RO were counted.

All distance measurements were performed in Fiji [45]. To measure the distance between the photoreceptors and bRPE cells, the distance between the RVRN-positive photoreceptor and the closest bRPE was measured at three points within each image. To measure the distance between two bRPE, the distance between a bRPE cell and the next closest bRPE cell was measured. For all distance measurements, the median was first calculated per image, and then for all three images per RO per condition.

The Kolmogorov–Smirnov test was used to test for normality [46]. To test for significance, a one-way ANOVA test, post hoc Tukey test, and Bonferroni correction for multiple comparisons were applied [39,40].

### 4.10. Matrigel Embedding and Analysis

A step-by-step guide to Matrigel embedding is shown in Figure S3. Matrigel GFR was used for all embedding experiments. Control ROs were not embedded. Embedded ROs were harvested after 4 h, 24 h, 48 h, or 1 week, and fixed in 0.1% glutaraldehyde (Carl Roth GmbH + Co. KG, Karlsruhe, Germany), 2% PFA for 30 min at RT. Sucrose infiltration, cryosectioning, and immunostaining were conducted as described previously [20].

Three images of the ONL were taken at 20x magnification with an Olympus Fluoview FV3000 confocal microscope. Only cryosections from the center of the RO were imaged and analyzed. RCVRN-positive IS and PRPH2- or ROM1-positive OS were manually counted using the cell counter plugin in Fiji [45]. The median was calculated for at least three images per RO per condition, and the Kolmogorov–Smirnov test was used to test for normality [46]. To test for significance, a one-way ANOVA test, post hoc Tukey test, and Bonferroni correction for multiple comparisons were performed [39,40].

## 5. Conclusions

Embedding retinal organoids in a basement membrane-like substance for 24 h protects photoreceptor outer segments from mechanical shearing forces. A temporary functional interaction between RPE cells and photoreceptors was indicated after embedding retinal organoids with iPSC-derived isogenic RPE.

## Data Availability

Not applicable.

## Supplementary Materials

The following supporting information can be downloaded at: https://www.mdpi.com/article/10.3390/ijms232314893/s1.
