# Supplementary material for: Transient Retention of Photoreceptor Outer Segments in Matrigel-Embedded Retinal Organoids
Source: Int J Mol Sci. 2022 Nov 28;23(23):14893. doi: 10.3390/ijms232314893 (PMC9739155; doi:10.3390/ijms232314893)
Supplement: Supplementary file 1 [file ijms-23-14893-s001.zip › ijms-1980403-supplementary.pdf]

International Journal of
Molecular Sciences

Enhancing Retention of Photoreceptor Outer Segments in Retinal Organoids

Patricia Berber, Sofiia Bondarenko, Lisa Michaelis, and Bernhard H. F. Weber

Supplemental Material including

- Figure S1. Schematic overview of the different methods used for co-culturing ROs and bRPE cells.
- Figure S2. Four co-culture techniques failed to induce robust interaction between bRPE and RO photoreceptors
- Figure S3. A step-by-step guide to Matrigel embedding

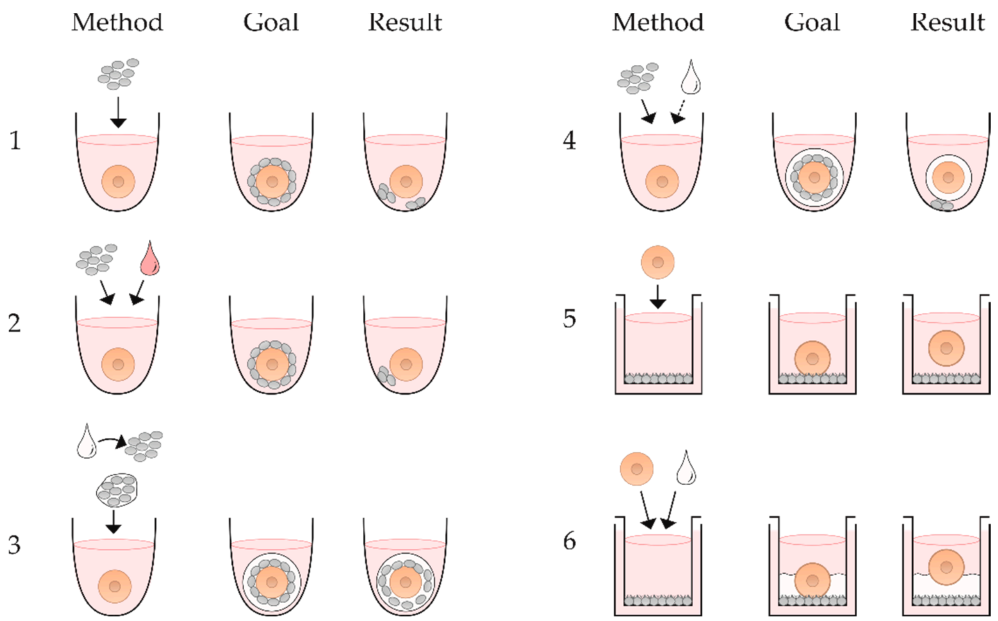

Figure S1. Schematic overview of the different methods used for co-culturing ROs and bRPE cells. (ROs are represented by light brown circles with a dark core; bRPE are represented by small grey circles; additives are represented by a pink droplet; Matrigel is represented by a white droplet.) (**1**) Co-cultures with dissociated bRPE revealed that most bRPE detached from the RO surface over time. (**2**) Co-cultures with dissociated bRPE were treated with ApInh or PrECM, which did not improve attachment. (**3**) bRPE were suspended in Matrigel and an RO was embedded in the bRPE–Matrigel mixture. A temporary functional interaction was observed, where the bRPE internalized RCVRN after 4 and 24 hours. Over time, the bRPE formed clusters within the Matrigel, and migrated away from the RO surface. (**4**) ROs were co-cultured with dissociated bRPE for 48 hours, and then suspended in Matrigel. No bRPE were observed on the RO surface after embedding. (**5**) In co-cultures with bRPE grown on Transwell filter inserts, the RO detached from the bRPE lawn. (**6**) Matrigel or Hydrogel was added to the co-cultures with bRPE grown on Transwell filter inserts, but the gels lifted the RO from the bRPE lawn. bRPE are represented by small grey circles.

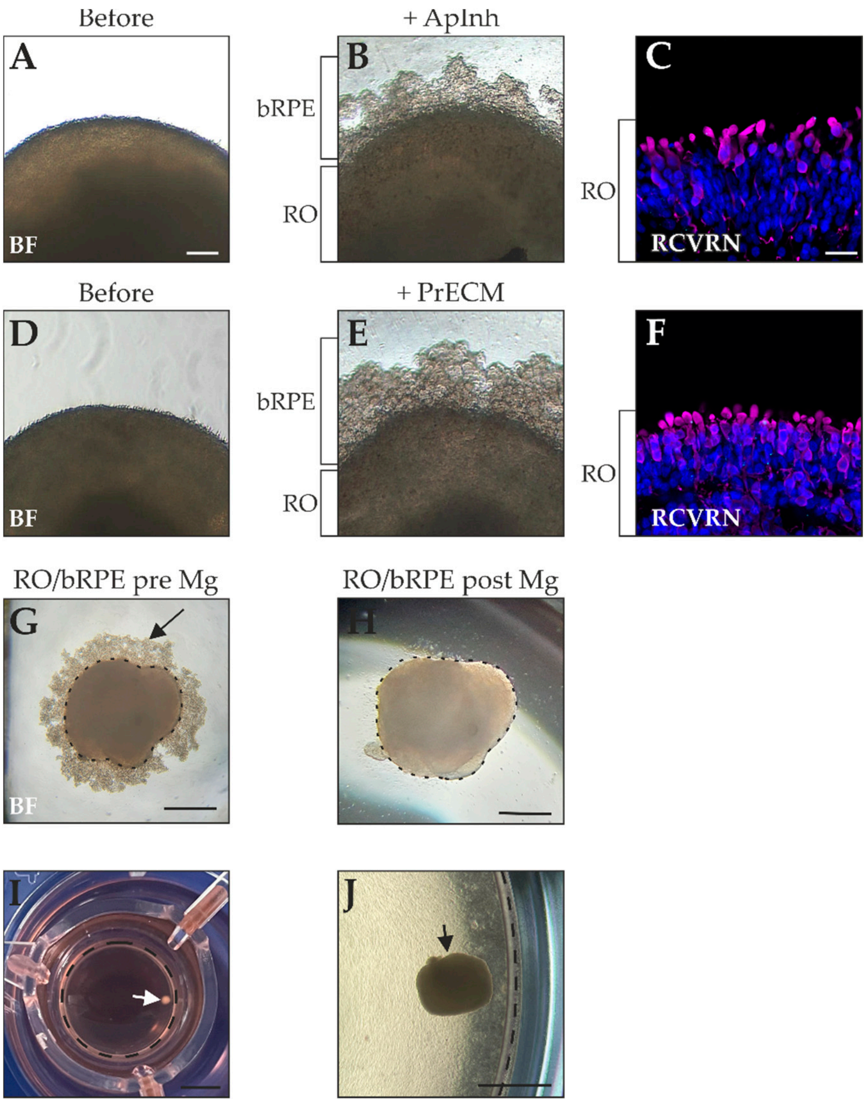

Figure S2. Four co-culture techniques failed to induce robust interaction between bRPE and RO photoreceptors. (**A**) Brightfield image of an RO prior to co-culture is shown. (**B**) Co-culture was induced according to Technique 2, described in Figure S1. ApInh (a supplement to reduce apoptosis), was added to an RO cocultured with dissociated bRPE, but it was not well-tolerated by the RO, as indicated by the darkening of the RO neuroepithelium. After 48 hours, bRPE were in close proximity to the RO. (**C**) ICC of the RO shown in B revealed RCVRN-positive photoreceptors but no bRPE. The bRPE seen in B detached from the RO during preparation for ICC. (**D**) Brightfield image of an RO prior to co-culture is shown. (**E**) Co-culture was induced according to Technique 2, described in Figure S1. PrECM (soluble components of the photoreceptor extracellular matrix) was added to a RO co-cultured with dissociated bRPE. After 48 hours, bRPE were in close proximity to the RO. (**F**) ICC of the RO shown in E revealed RCVRN-positive photoreceptors but no bRPE. The bRPE seen in E detached from the RO during preparation for ICC. (**G**) Co-culture was induced according to Technique 4, described in Figure S1. An RO (indicated with a dashed line) was co-cultured with dissociated bRPE (arrow). (**H**) After the coculture shown in G was embedded in Matrigel, no bRPE remained around the RO (indicated with a dashed line). (**I**) Co-culture was induced according to Technique 5, described in Figure S1. bRPE were grown on a Transwell filter insert (indicated by a dashed line) and an RO (white arrow) was plated on the filter. This image is not magnified. (**J**) The co-culture shown in I is shown at a 2x magnification. Although the RO (black arrow) initially weakly attached to the bRPE lawn (indicated by a dashed line), it invariably detached despite careful handling. Scale bars: (A, B, D, E) 100 µm, (C, F) 20 µm, (G, H) 500 µm, (I) 3 mm, (J) 1 mm.

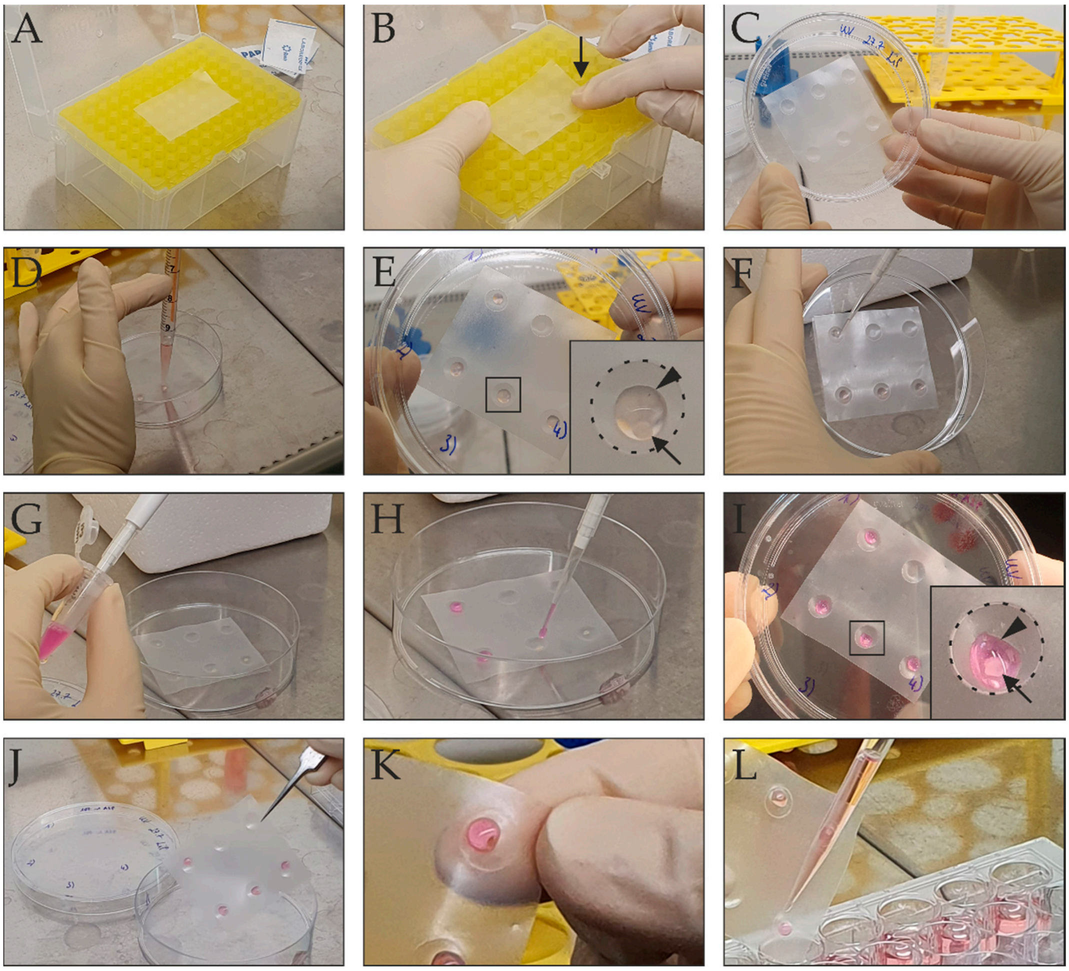

Figure S2. A step-by-step guide to Matrigel embedding. (**A**) Parafilm™ molds were created by placing a square of Parafilm™ on an empty filter tip box (size 10 µl - 100 µl) and (**B**) pressing down to form an indentation. (**C**) Parafilm™ molds were transferred to a 10 cm tissue culture dish and sterilized by placing it under a UV light source for 30 min. (**D**) Each RO was carefully placed within one indentation, while transferring as little media as possible. (**E**) An RO (arrow) within an indentation (dashed circle) and a drop of media (arrowhead) is shown. (**F**) The media were carefully removed without infringing RO integrity. (**G**) 20 µl Matrigel (kept on wet ice prior to use) was (**H**) dropped onto the RO. (**I**) An RO (arrow) within an indentation (dashed circle) and Matrigel droplet (arrowhead) is shown. (**J**) The ROs in Matrigel were incubated at 37 ºC for 30 min. (**K**) The back of each Parafilm™ indentation was pressed to facilitate RO transfer. (**L**) ROs were transferred to single wells of a 24-well plate by very gently washing the embedded RO off the Parafilm™ with media. Rough pipetting was avoided as it increases the risk of damaging the Matrigel casing.
